# Single session high definition transcranial direct current stimulation to the cerebellum does not impact higher cognitive function

**DOI:** 10.1371/journal.pone.0222995

**Published:** 2019-10-10

**Authors:** Ted Maldonado, James R. M. Goen, Michael J. Imburgio, Sydney M. Eakin, Jessica A. Bernard

**Affiliations:** 1 Department of Psychological and Brain Sciences, Texas A&M University, College Station, Texas, United States of America; 2 Texas A&M Institute for Neuroscience, Texas A&M University, College Station, Texas, United States of America; University Medical Center Goettingen, GERMANY

## Abstract

The prefrontal cortex is central to higher order cognitive function. However, the cerebellum, generally thought to be involved in motor control and learning, has also been implicated in higher order cognition. Recent work using transcranial direct current stimulation (tDCS) provides some support for right cerebellar involvement in higher order cognition, though the results are mixed, and often contradictory. Here, we used cathodal high definition tDCS (HD-tDCS) over the right cerebellum to assess the impact of HD-tDCS on modulating cognitive performance. We predicted that stimulation would result in performance decreases, which would suggest that optimal cerebellar function is necessary for cognitive performance, much like the prefrontal cortex. That is, it is not simply a structure that lends support to complete difficult tasks. While the expected cognitive behavioral effects were present, we did not find effects of stimulation. This has broad implications for cerebellar tDCS research, particularly for those who are interested in using HD-tDCS as a way of examining cerebellar function. Further implications, limitations, and future directions are discussed with particular emphasis on why null findings might be critical in developing a clear picture of the effects of tDCS on the cerebellum.

## Introduction

In recent decades there has been an increase in work investigating the cerebellum in non-motor function (for a review see [1]). The application of non-invasive neuromodulation such as transcranial direct current stimulation (tDCS) has made it possible to better understand non-motor functions of the cerebellum. This technique is especially informative, as one is able to increase or decrease the influence of cerebellar behavior to investigate the relative necessity of the cerebellum in task performance. Critically, evidence suggests that cerebellar tDCS has modulated task performance in several cognitive domains [2,3], though results have been mixed, possibly due to variability in methodologies, and stimulation approaches that may not be optimal for targeting the cerebellum.

Traditionally, the cerebellum was thought to be primarily involved in motor function [4] and motor learning (c.f. [5]). However, there is a growing literature suggesting that the cerebellum also contributes to non-motor behavior [6–13]. Notably, within the cerebellum there appears to be a segregated functional topography (e.g. [14,15]). That is, the anterior lobules and lobules VIIIa and VIIIb are implicated in motor functioning, whereas the remaining posterior lobules are associated primarily with cognitive functioning [5,14,16]. Within the posterior lobules, imaging work has consistently demonstrated activations in the while completing working memory [10,15,17–19], updating [20], planning [21], and shifting tasks [22], with activations primarily found in the right cerebellum [7,15,23], particularly for tasks involving language and verbal abilities. Further, some lesion work has also examined executive control [19], suggesting that the cerebellum might play a role in inhibition [24] and shifting ability [25]. Globally, cerebellar lobular volume has predicted cognitive function [5,26]. On the circuit level, research has found specific cerebello-thalamo-cortical closed loop circuits in both non-human primates [27–29] and humans [30–32], with recent work suggesting resting state co-activations between the PFC and cerebellum predicted performance on learning and executive function tasks [33].

While there is a growing evidence implicating the cerebellum in cognitive function, the extent to which the brain relies upon this region for the performance of cognitive tasks and computations remains relatively underexplored. Patient work has suggested some degree of necessity of this region, as individuals with lesions to the cerebellum show marked deficits in cognitive functions [9, 34–37]. In addition, work using transcranial magnetic stimulation (TMS), has also suggested that cerebellar function was key for optimal performance. Brief disruption of cerebellar function negatively impacted working memory performance [38,39]. Further, tDCS holds promise in this area of research. tDCS is a noninvasive form of neuromodulation that increases (anodal) or decreases (cathodal) neural activity through the use of a small amount of electric current in order to examine brain regions in pseudo isolation [40]. Behaviorally, this generally results an increase or a decrease in motor [41] or non-motor functioning [42], after cortical stimulation. However, with cerebellar tDCS the evidence is mixed as to whether performance modulation is polarity-specific [2].

To date, tDCS has been used in conjunction with a variety of behavioral tasks, though the work examining the role of the cerebellum in cognition is relatively limited (for a review see [2]) and mixed. Pope and Miall (2012) found that cathodal tDCS over the right cerebellum improved performance on working memory tasks, particularly as cognitive demands increased, speculating that cathodal cerebellar tDCS dis-inhibits the prefrontal cortex (PFC; [43]). Conversely, Boehringer and colleagues (2013) investigated the cerebellum in verbal working memory using cathodal stimulation to the right cerebellum before completing a forward and backward digit span [44]. This resulted in performance decrements the authors believed resulted from tDCS negatively impacting communication between the cerebellum and PFC. Further, Ferrucci et al. (2008) found that *both* anodal and cathodal stimulation over the right cerebellum impaired the practice dependent improvement in reaction time during a Sternberg task, such that reaction times did not differ with two successive completions of the Sternberg task after stimulation. However, cathodal tDCS delivered to the PFC did impact performance immediately after stimulation [45]. Most recently, Spielmann et al., (2017) looked to replicate and extend existing work by investigating cathodal stimulation over the right cerebellum with cognitive tasks. Notably, they were unable to replicate previous findings. However, the authors speculated that cathodal stimulation might have long term effects on cognition. That is, participants who received cathodal stimulation saw reduced performance and increased variability in task performance when tested one week later [46]. Further, there are some instances in which stimulation has no effect at all on task performance [47–50]. Thus, while there is evidence to indicate a role for the right cerebellum in cognitive function, the results to date using traditional tDCS methodologies remain mixed as the results are not polarity specific and the effects of stimulation do not always manifest at predictable time points.

Taken together, there is a vibrant and growing literature implicating the cerebellum in cognitive processes. However, the specific role of the cerebellum in cognition is not clearly understood, and results using standard tDCS approaches are mixed. While tDCS has the potential to advance our understanding of the role of the cerebellum in non-motor processes, and further augment the TMS and lesion literatures, the mixed results in this regard make interpreting the existing findings challenging. With that said, methodological approaches may be a particularly important consideration. Traditionally, tDCS has used a two-paddle system such that current is sent from one electrode and is received by a second electrode with limited finesse in targeting a brain region of interest [51]. Large electrode pads have been used (typically 5x5 or 5x7 cm), and these result in poor targeting of the underlying cerebellar regions. Further, anatomical landmarks are the primary means of localization of the cerebellum, potentially further negatively impacting targeting. The advent of high definition tDCS (HD-tDCS) allows increased precision in regional targeting by taking advantage of multiple smaller electrodes that, when used in conjunction with modelling software [52,53], can optimally direct current through the scalp and cortex to better target a region of interest. Such improved targeting of stimulation may provide a better approach to cerebellar stimulation, particularly given that modelling software allows for the specific targeting of the lateral posterior cerebellum [52,53], which as noted, is part of networks with the PFC and has been implicated in cognitive processing [27,32,54,55].

Thus, the current study took advantage of HD-tDCS to investigate cerebellar contributions to executive function [56–58]. We hypothesized that the cerebellum plays an integral and necessary role in cognitive functioning, and as such, predicted that cathodal stimulation would negatively impact behavioral performance. Temporarily reducing function to negatively impact behavior is necessary to understand the role the cerebellum plays in cognition. Cerebellar tDCS results in temporary “virtual lesions”, paralleling the approach use in the transcranial magnetic stimulation (TMS) literature [59]. Thus, when we apply cathodal tDCS, it is to temporarily reduce the influence the cerebellum might have on task performance, which allows us to understand the relative necessity of cerebellar contributions to performance, thus improving our understanding of cerebellar contributions to cognition. Because of past work implicating the right cerebellum in working memory and language functions [7,23,43–45,60], tDCS was focused on the right hemisphere of the cerebellum given the tasks employed here.

## Methods

### Participants

Thirty-four right handed Texas A&M University undergraduate students enrolled in this study for partial course credit in an Introduction to Psychology course. Ten participants were removed from the analysis because they did not complete the second visit (n = 9) and/or because of a computer error that resulted in incomplete data sets (n = 1). Thus, 24 right handed participants (12 female) ages 18 to 25 (*M* = 19.04 years, *SD* = 1.60) were used in the final analysis. All procedures completed by participants were approved by the Texas A&M University Institutional Review Board and conducted according to the principles expressed in the Declaration of Helsinki.

### Procedure

Data were collected across two visits as two different stimulation types (cathodal and sham) were used in the experiment. Further, two visits reduce the chance the participant learns which stimulation condition they are participating in and minimizes any immediate practice effects from the task paradigms. Data suggest that the effects of tDCS may last up to 90 minutes [61], and this one-week interval ensures that all effects of tDCS have worn off. During visit one, after a written consent form was signed, participants completed a basic demographic survey and the Edinburgh Handedness Survey [62] to confirm right-handedness. After completing the questionnaires, participants were prepared for tDCS (see below for details). Once tDCS was complete, participants then completed computerized Stroop [63] and Sternberg tasks [64] in a pre-determined random order (for more details, see below). The second visit, completed seven days later for all participants, was identical to the first, with the exception of the polarity of the stimulation, such that participants who received sham in the first visit received cathodal stimulation in the second or vice versa. Stimulation was administered in a single-blind manner, wherein participants were blinded to the stimulation type across the two sessions. Critically, the order of stimulation across the two sessions was randomly assigned and counterbalanced across participants.

#### tDCS stimulation parameters

**Fig 1:**
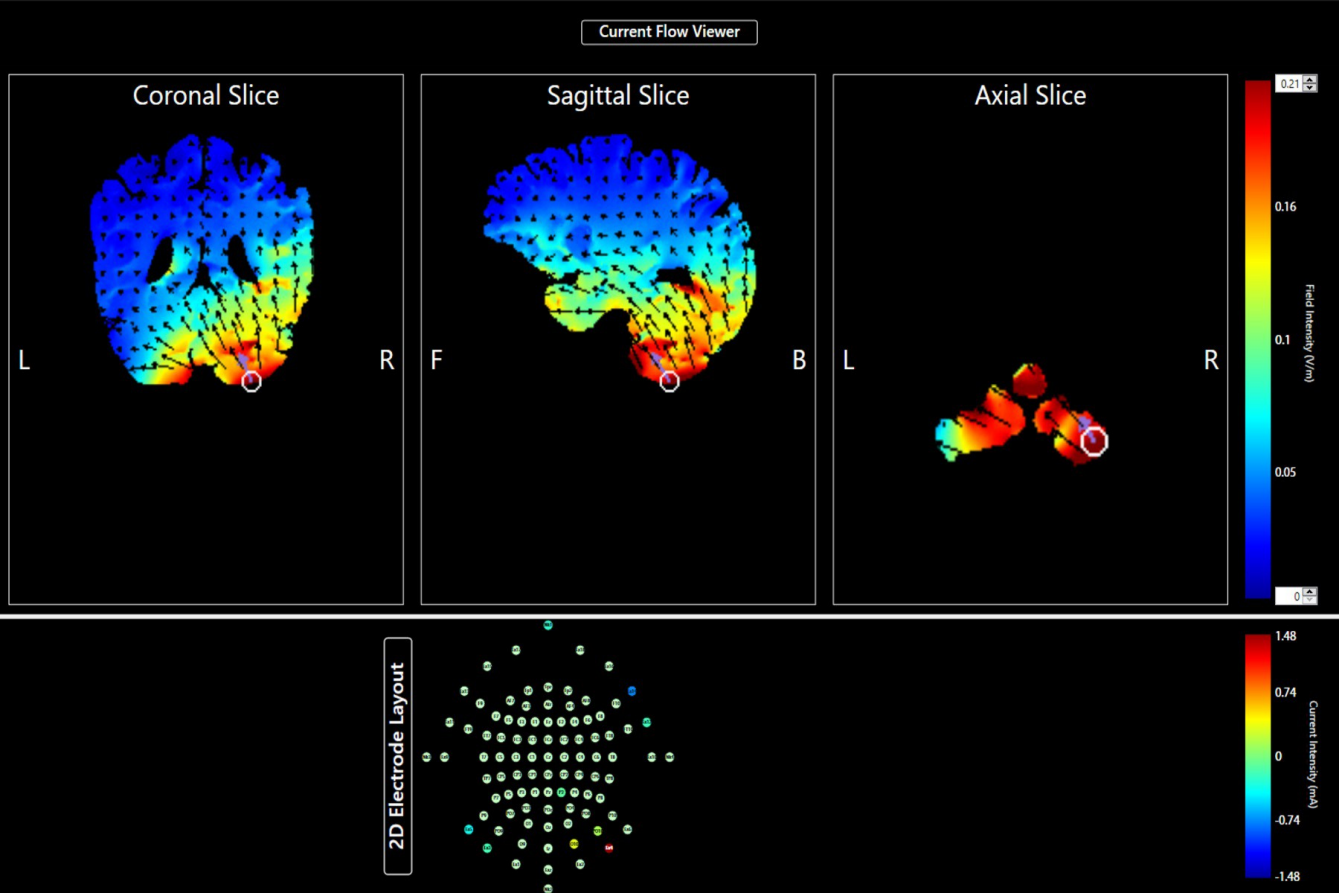
Modelled current flow and intensity (in V/m ranging from 0 to .21) montage using Soterix targeting software. **cooler colors indicate lower intensities while warmer colors indicate higher intensities.** *Note*. L = Left, R = Right, F = Front, B = Back.

Participants were first fitted with a cap with electrode holders with positioning that followed the 10–20 electrode system. Specifically, the diameter of the participant’s head was measured to ensure the correct cap size was used. The cap was centered over Cz and held into place with a chin strap. Electrodes were placed in pre-determined locations to optimize stimulation to the right cerebellum. In order to take advantage of our Soterix MxN HD-tDCS system, we used 9 electrodes, wherein one electrode was the return electrode, and the other 8 provided stimulation. Such a montage increases the focality of the current to the target area, and minimizes the stimulation of neighboring regions. Typically, there is a predominately positive or a predominately negative current applied to the scalp that is localized using multiple electrodes applying a current opposite of the central electrodes [65–68]. In the present study, the three negative electrode sites set the cathodal polarity, and the six positive currents confine the current to increase focality to the right cerebellum (See Table 1), with one electrode used as a return. Electrode placements and intensities (Table 1) for an adult head were determined by entering the number of electrodes and the desired stimulation location into Soterix Neurotargeting HD-Targets Software [52,53]. The software has specific algorithms that derive the optimal stimulation intensities that will ensure that the desired region is specifically targeted. Electrodes were fixed to the head using a specialized cap or specially designed elastic straps which extended the cap beyond a 64-channel montage to allow current to successfully reach the cerebellum. Fig 1 presents the modelled current flow targeting the right lateral posterior cerebellum.

**Table 1:** Current intensities (in mA) and locations for cerebellar cathodal stimulation.

	Cerebellar Cathodal Stimulation	
	Location	Current
**1**	P2	0.1135
**2**	PO10	-0.1551
**3**	O10	-0.3618
**4**	Ex3	0.1956
**5**	Ex4	-1.4832
**6**	Ex5	0.4066
**7**	Ex12	0.22
**8**	Ex14	0.7886
**C**	Nk1	0.2757

The stimulation session began with a brief attenuation period in which all electrodes were set at 0.1 mA for one minute to ensure a good connection with the scalp and that the appropriate impedance levels for stimulation (less than 100 KOhm) were reached. For any electrode where impedance was above 100 KOhm, additional gel was added, and impedance was checked again. Once all impedances were in the accepted range, participants were placed in a comfortable chair for a 20-minute stimulation session, using the current values presented in Table 1. During cathodal stimulation, the currents gradually increased for 30 seconds, maintained intensity for 20 minutes, and gradually decreased for 30 seconds. During sham conditions, the currents gradually increased for 30 seconds until currents were reached, then gradually decreased for 30 seconds directly before and after the 20-minute session. There was no additional stimulation during the 20-minute session. During stimulation conditions, participants were asked to relax, so that external stimuli would not influence the effectiveness of the stimulation. In total, participants received 2 mA of stimulation, consistent with previous cerebellar research [51]. Once stimulation was completed, impedances were checked again to ensure the appropriate connections were maintained through the duration of the stimulation. There were two instances where impedances were not recorded or high, but they were both during sham stimulation so no further actions were taken. Stimulation was followed by the completion of two cognitive task paradigms.

#### Cognitive paradigms

The order of the following tasks was counterbalanced. In total, the tasks took 30 to 35 minutes to complete, including time for instructions. We chose to use the Stroop [63] and Sternberg [64] tasks. To date, the majority of cerebellar tDCS work has examined verbal working memory [3]. Thus, in order to support and replicate past work, participants completed a verbal working memory task (Sternberg). In order to extend the current literature, we also included an inhibition task (Stroop).

**Stroop**. The Stroop task [63] was administered via computer using a preprogrammed script from Experiment Factory [69]. Experiment Factory is an online repository that publishes experiments with the intent of improving replicability. Participants were instructed to identify the ink color for a word on the computer screen. In 50% of trials, the ink color and the word were congruent, such that the word ‘Green’ was written in green ink. In the remainder of the trials, the ink color and word were incongruent such that the word ‘Green” might be written in blue ink. Participants were told to respond to the ink color by pressing the ‘R’ key for red ink, the ‘B’ key for blue ink and the ‘G’ key for green ink. Participants were instructed to respond as quickly and accurately as possible. Stimuli were shown for 1500 milliseconds, followed by 500 milliseconds of feedback which indicated whether the response was correct, incorrect, or if the participant needed to respond faster. Participants completed 120 trials. Dependent variables were average reaction time and accuracy. Additionally, the Stroop effect, or the interference experienced when naming the ink color during incongruent trials, was also used as a dependent variable.

**Sternberg**. The Sternberg Task [64] was administered via computer using

Presentation Software (Version 18.0, Neurobehavioral Systems, Inc., Berkeley, CA, www.neurobs.com). Three load-levels were used: either one, three, or five items, representing low, medium, and high loads, respectively. At the start of each trial, capitalized letters were presented for 5 seconds. The number of letters presented varied based on the load condition. Following the presentation of the capitalized letters, participants were presented with individual lower-case letters, one at a time, presented at a rate of one letter per second. Participants were instructed to decide whether the letter presented to them was one of the original letters presented at the beginning of the trial and respond via button press for each letter. Participants completed two blocks for each load level for a total of six blocks. Each block had 25 trials each, for a total of 150 trials. Dependent variables were average reaction time and accuracy.

### Data processing and analysis

Statistical analyses were completed in R [70] using the “ez” package [71]. Shapiro-Wilk normality tests were completed for each dependent variable assessed. Log transformations were applied to dependent variables that had non-normal distributions. Further, data were checked for outliers by using three standard deviations above and below the mean as an *a priori* threshold. Both the Sternberg (S1 Table) and Stroop (S2 Table) task data sets can be found on the Open Science Framework website (https://osf.io/rcu25/).

Data from the Stroop task were analyzed using a 2 (stimulation type: sham v cathodal) x 2 (congruency: congruent v incongruent) repeated measures ANOVA to investigate the influence of stimulation on Stroop performance. This analysis was run three times, once for each dependent variable: reaction time, accuracy, and the Stroop effect. Similarly, a 2 (stimulation type: sham v cathodal) x 3 (load: high v medium v low) repeated measure design was used to assess the effect of stimulation on Sternberg performance. This analysis was run for both dependent variables: reaction time and accuracy. For dependent variables for which a log transform did not normalize the distribution, nonparametric methods were using the *nparLD* package [72,73].

All results were evaluated with a statistical threshold wherein p < .05 was used as the cut-off for significance. Significant effects were followed up with Tukey’s HSD post-hoc tests for normally distributed parameters and paired Wilcoxon Sum-Rank Tests for non-normal parameters. Generalized effect sizes (*η*^*2*^_*G*_) were used to quantify the size of each effect, as they are better suited to describe repeated measure designs [74]. As such, an effect size of 0.02, 0.13, and 0.26 represent small, medium, and large effect sizes, respectively.

## Results

### Stroop task

Descriptive statistics are presented in Table 2. A Shapiro-Wilk normality test was conducted on each dependent variable (reaction time, accuracy, and Stroop effect). The test revealed that the reaction time (*W* = 0.976, *p* = 0.08) and the Stroop effect (*W* = 0.963, *p* = 0.13) variables were normally distributed, but accuracy (*W* = 0.759, *p* < .001) was not, which was supported with a density plot showing a strong skew to the left. Thus, accuracy was log transformed. Following the log transform, accuracy was still not normally distributed (*W* = 0.744, *p* < .001), thus non-parametric methods were used on this variable.

**Table 2:** Descriptive statistics for the Stroop and Sternberg task. Reaction time is reported in milliseconds and accuracy is reported in percent correct.

	**Stroop**			
	Stim	Congruency	Mean	SD
**Reaction Time (ms)**	Real	Congruent	661.34	61.36
	Real	Incongruent	780.72	76.12
	Sham	Congruent	669.91	82.52
	Sham	Incongruent	768.74	110.08
**Accuracy**	Real	Congruent	0. 99	0. 01
	Real	Incongruent	0. 96	0. 03
	Sham	Congruent	0. 99	0. 02
	Sham	Incongruent	0. 95	0.05
	**Sternberg**			
	Stim	Load	Mean	SD
**Reaction Time (ms)**	Real	High	624.13	59.15
	Real	Low	491.74	52.44
	Real	Medium	564.11	52.93
	Sham	High	630.03	60.64
	Sham	Low	482.89	70.31
	Sham	Medium	574.97	64.75
**Accuracy**	Real	High	0.87	0.09
	Real	Low	0.95	0.06
	Real	Medium	0.94	0.04
	Sham	High	0.86	0.11
	Sham	Low	0.90	0.16
	Sham	Medium	0.93	0.05

**Fig 2:**
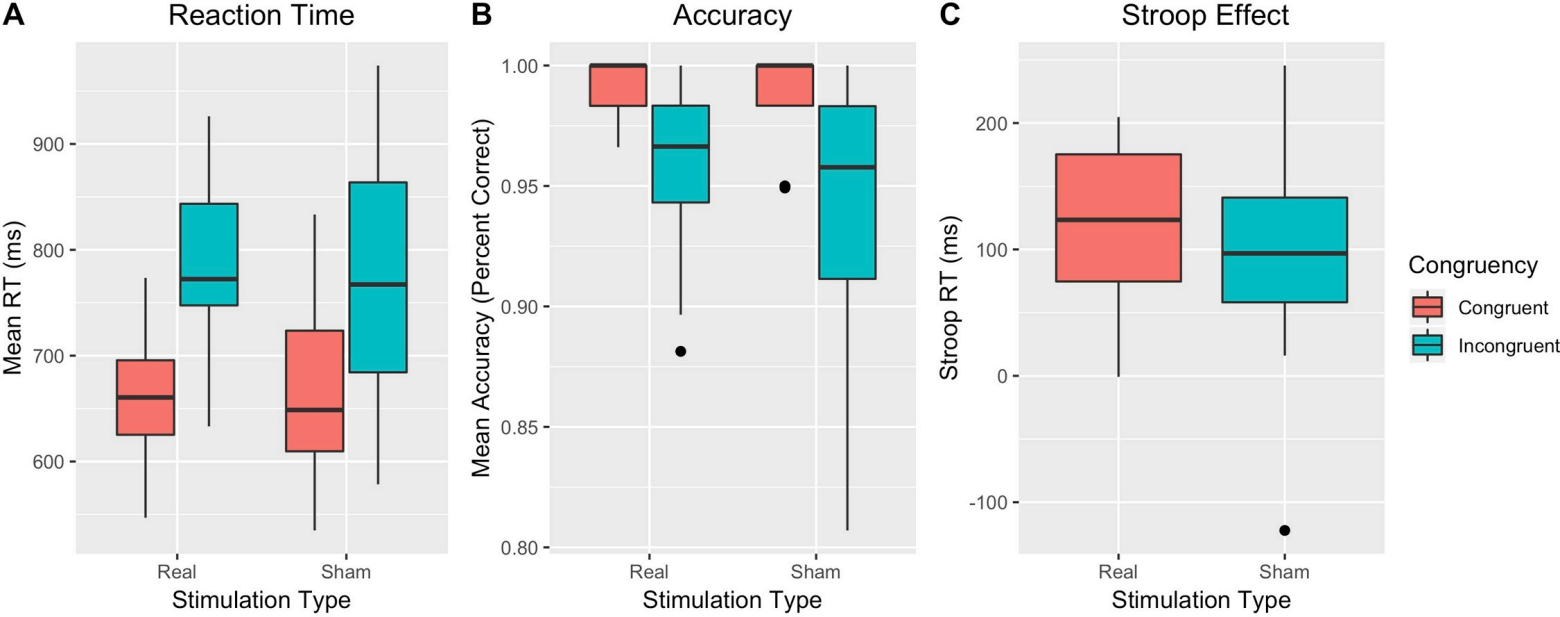
(A) Reaction times on the Stroop task by stimulation type; (B) Percent accuracy on the Stroop task by stimulation type; (C) Stroop effect for reaction time.

When examining RT (Fig 2), there was a main effect of congruency [*F*(1, 23) = 112.11, *p* < .001, *η*^*2*^_*G*_ = .304], such that participants had slower response time on incongruent trials relative to congruent trials. There was no main effect of stimulation [*F*(1, 23) = .0139, *p* = .907, *η*^*2*^_*G*_ = .000], nor was there a significant congruency x stimulation interaction [*F*(1, 23) = 1.39, *p* = .250, *η*^*2*^_*G*_ = .004].

When examining accuracy (Fig 2), non-parametric tests were used. A Wald Chi-Squared test suggests there was an effect of congruency [χ^*2*^ *= 41*.*53*, *df = 1*, *p*<0.001], such that participants were more accurate on congruent than incongruent trials. There was no effect of stimulation [χ^*2*^ *= 0*.*03*, *df = 1*, *p* = 0.864], nor was there a significant congruency x stimulation interaction [χ^*2*^ *= 0*.*23*, *df = 1*, *p* = 0.628]. Lastly, there was no effect of stimulation on the Stroop effect [*F*(1, 23) = 1.39, *p* = .250, *η*^*2*^_*G*_ = .02].

### Sternberg working memory task

A Shapiro-Wilk normality test was conducted on each dependent variable (reaction time and accuracy). The test revealed that the reaction time (*W* = 0.991, *p* = 0.49) variable was normally distributed, but accuracy (*W* = 0.762, *p* < .001) was not. Additionally, there were four accuracy scores three standard deviations below the mean. Following the removal of the outliers (*W* = 0.826, *p* < .001) and after the data was log transformed (*W* = 0.686, *p* < .001), accuracy still had a non-normal distribution. Thus, non-parametric methods were used on this variable.

**Fig 3:**
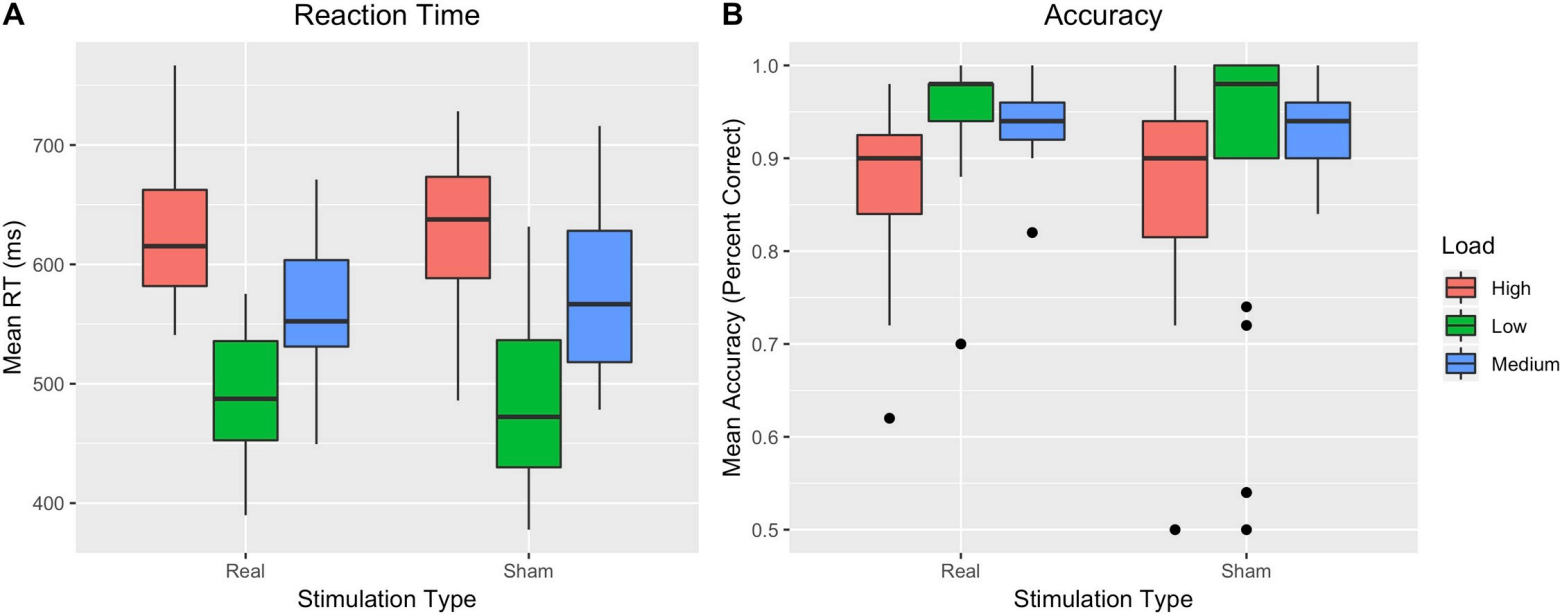
(A) Reaction times on the Sternberg task by stimulation type; (B) Percent accuracy on the Sternberg task by stimulation type.

When examining reaction time (Fig 3), there was a main effect of load [*F*(2, 46) = 217.31, *p* < .001, *η*^*2*^_*G*_ = .485] such that high, medium, and low load were all significantly different from each other (*p*s < .001) when using a post-hoc Tukey’s test. There was no main effect of stimulation type [*F*(1, 23) = .07, *p* = .793, *η*^*2*^_*G*_ = .000], nor was there a significant interaction between stimulation type and load [*F*(2, 46) = 1.18, *p* = .316, *η*^*2*^_*G*_ = .005].

When examining accuracy using non-parametric methods (Fig 3), a Wald Chi-square test suggested there was a significant effect of load [χ^*2*^ *= 40*.*76*, *df = 2*, *p*<0.001]. A post-hoc Wilcoxon Sum-Rank Tests with Bonferroni-corrected p-values found accuracy under high load was significantly worse than under medium and low load (*p*s < .002), though medium and low load were not significantly different (*p* = .247). There was no effect of stimulation type [χ^*2*^ *= 0*.*47*, *df = 1*, *p* = 0.493], nor was there a significant stimulation type by load interaction [χ^*2*^ *= 0*.*60*, *df = 2*, *p =* 0.741]. For both the Sternberg and Stroop task, transformed results were largely consistent with the analysis conducted on the un-transformed data.

### Bayesian analyses

Generally, a failure to reject the null hypothesis indicates that there is no significant difference between experimental conditions. Thus, in the current work, traditional null-hypothesis statistical testing does not allow us to concretely conclude that cathodal stimulation over the cerebellum had no effect on cognitive performance. This testing simply helps us understand the probability that our results are significant. However, Bayesian statistics can be applied to the likelihood of the data under the null hypothesis (i.e. stimulation did not have an effect on performance) versus the alternative hypothesis (i.e. stimulation did have an effect on performance). This is numerically represented by a Bayes Factor (BF_10_), which is interpreted similarly to an odds ratio. Classically, a Bayes factor greater than one indicates support for the alternative hypothesis. Alternatively, a Bayes factor less than one indicates support for the null hypothesis [75]. Therefore, BF_10_ = 10 indicates the data are 10 times more likely under the alternative hypothesis than under the null hypothesis, whereas a BF_10_ = 0.2, indicates the data are 5 times more likely under the null hypothesis. We used JASP [76] to calculate Bayes Factors for all repeated measures ANOVAs conducted above to see which hypothesis was best predicted by the data.

The analyses showed that for reaction time and accuracy for both the Stroop and Sternberg tasks, the best model only included a main effect of load (Sternberg) or congruency (Stroop) with BF_10_ over 80. The BF_10_ for stimulation were between .2 and .5, suggesting moderate support for the null hypotheses, that is, that stimulation likely did not affect performance. The same pattern held for the Stroop effect (BF_10_ = .399). Thus, Bayesian analyses conducted for each model provide modest support the hypothesis that cerebellar HD-tDCS is statistically unlikely to modulate reaction time or accuracy on the Stroop or Sternberg tasks.

### Drift diffusion model

To assess effects of stimulation on latent decision-making processes, a drift diffusion model (DDM; [77]) was fit to trial-level data in each task using fast-dm [78]. DDMs allow for the estimation of a number of parameters relevant to two-choice psychology tasks, including drift rate (the signal-to-noise ratio during task performance), nondecision time (a portion of the RT unrelated to active processing of the stimulus, such as visual processing, motor response and task set preparation), and response caution (a quantification of speed-accuracy tradeoff). DDMs may also yield a bias parameter that quantifies a participant’s bias prior to stimulus presentation; however, because our data was coded as either correct or incorrect, the candidate models all assumed no bias (fixed at 0.5) as subjects would not have had knowledge of which response is correct prior to stimulus presentation. Drift rate, response caution, and nondecision time were all allowed to vary by stimulation condition and task condition (load or congruency) to assess the effects of each on the parameters.

Following fast-dm recommendations, models were fit using the maximum likelihood method (because each participant performed 50 trials of each condition). Notably, models fit using the maximum likelihood method are not robust to outliers; therefore, trials on which RT was lower than 150 ms or greater than 3 standard deviations from the mean were removed prior to model fitting. The resulting parameters from these models were then checked for normality; normally distributed parameters were analyzed using 2x2 within-subjects ANOVAs while non-normally distributed parameters were analyzed using the nonparametric Wald test [73] from the *nparLD* package [72]. Significant effects were followed up with Tukey’s HSD post-hoc tests for normally distributed parameters and paired Wilcoxon Sum-Rank Tests with Bonferroni-corrected *p*-values for non-normal parameters.

For the DDM fit to the Stroop task, there was a significant main effect of congruency [χ^2^(1) = 24.24, *p* < .001] on drift rate such that the signal-to-noise ratio during decision making was better during congruent trials (*Med*. = 3.82) than incongruent trials (*Med*. = 2.48). There was no main effect of stimulation condition nor interaction between congruency and stimulation condition, *p*s > .14, on drift rate. There were no significant main effects of stimulation condition or congruency, nor significant interactions, on response caution (*a*) or nondecision time (*t0*) on the Stroop task, *p*s > .32.

For the model fit to the Sternberg task, there was a significant main effect of load on drift rate [χ^2^(2) = 168.94, *p* < .001]. The signal-to-noise ratio during decision making was significantly higher in the low load condition (*Med*. = 3.93) than the medium load condition (*Med*. = 3.26; *p* = .02), which in turn was significantly better than the signal-to-noise ratio in the high load condition (*Med*. = 0.26; *p* < .001). There was a significant main effect of load on response caution [χ^2^(2) = 0.12, *p* = .02]. Follow-up tests revealed that participants were significantly more cautious (greater emphasis on accuracy over speed) in the low load condition (*Med*. = 1.06) than the high load condition (*Med*. = 0.90; *p* = .01). Response caution in the medium load condition (*Med*. = 0.97) did not significantly differ from either of the other two load conditions, *p*s > .30. Finally, nondecision times varied significantly by load [*F*(2, 46) = 65.58, *p* < .001]. Nondecision times were greater for the high load condition (*M* = 0.46, *SD* = 0.07) than the medium load condition (*M* = 0.43, *SD* = 0.06; *p* = .03), which was greater than for the low load condition (*M* = 0.35, *SD* = 0.04; *p* < .001). The progressive increase in nondecision time with increases in working memory load might reflect greater periods of time spent loading working memory representations prior to decision making. There was no interaction between stimulation and load, nor main effect of stimulation, on any of the parameters (*p*s ≥ .10).

## Discussion

The literature implicating the cerebellum in higher-order cognition is growing, though the vast majority of this work has been correlational in nature due to the limitations of fMRI and resting state connectivity. There are relatively few studies taking advantage of patient populations given the challenges inherent to this work (e.g [9,79,80]), and TMS (e.g. [38,39], though these literatures suggest that the cerebellum is critical for optimal performance. tDCS provides an interesting alternative method to investigating the relative necessity of the cerebellum in cognitive function given the built-in sham option. However, work to date using traditional tDCS methods has been mixed with respect to non-motor tasks [43–46]. Here, we took advantage of the improved targeting that comes with HD-tDCS to stimulate the lateral posterior cerebellum, which is known to be engaged in cognitive processing and has both functional and structural connections with the prefrontal cortex [33]. Performance on a verbal working memory task (Sternberg) and an inhibition task (Stroop) was quantified after both sham and cathodal (inhibitory) stimulation. While we initially predicted that cathodal stimulation would have a negative impact on performance consistent with the general literature suggesting the inhibitory nature of cathodal stimulation [81,82] our findings demonstrated no effect of stimulation on performance of either task.

Overall, our results showed the expected cognitive demand effects. Specifically, when examining Stroop data, we found that participants responded slower, and less accurately to incongruent than to congruent trials. Critically, when examining how stimulation affected performance, we found that cathodal stimulation did not significantly affect reaction time or accuracy. Similarly, when we examined Sternberg task performance, we found that reaction time was slowest for high load, followed by medium load, and fastest for low load. We also found that accuracy was lowest for high load, followed by medium and load loads, which had similar accuracies. Again, cathodal stimulation did not significantly affect task performance. This was supported by Bayesian analyses that suggested an effect of stimulation was not likely and drift diffusion models that suggest stimulation does not affect drift rate, response caution, or nondecision time. This was the case for both tasks.

While the expected congruency and load manipulations emerged in the Stroop and Sternberg tasks, respectively, there was no effect of cerebellar cathodal stimulation on performance across any of our metrics. This is counter to previous research demonstrating effects of cerebellar stimulation on non-motor task performance [43–46,58], but is not the first null finding in the literature. van Wessel, Verhage, Holland, Frens, & van der Geest (2016) conducted a within subjects study in which twelve participants underwent anodal, cathodal, and sham stimulation over the cerebellum before the completion of an n-back task and found load effects but no effect of stimulation [47]. The authors noted a small sample size and size of electrode as possible limitations. Recently, Verhage, Avila, Frens, Donchin, and van der Geest (2017), completed a larger *between* subjects design in which anodal (n = 20) or sham (n = 19) stimulation was applied over the right cerebellum before an implicit categorization task and did not find effects of anodal stimulation on task performance [49]. Similar between subjects studies applied either anodal, cathodal, or sham stimulation over the cerebellum of 30 participants (10 per stimulation condition) and again found no effect of stimulation in an implicit categorization learning task [50] or a probabilistic classification learning task [48]. Though these studies found similar results to those described in the current work, two key differences exist between previous study designs and the one currently used. First, the current study used a *within* subjects design that had 24 participants per stimulation type, compared to 10–12 participants typically seen in previous studies. Second, our work here used HD-tDCS, instead of the traditional two electrode system. Critically, even with our sample size, which is relatively large in comparison to past investigations reporting null findings, as well as the improved targeting of HD-tDCS with smaller electrodes (two key limitations noted by [47]), we still found null effects. Thus, cathodal HD-tDCS did not impact behavior on two well-known and robust cognitive paradigms.

In the discussion of their null findings, Steiner and colleagues (2016), suggested several limitations such as task dependent effects of tDCS, electrode size and placement, and ceiling effects as reasons for their null findings, which might also be relevant to the current study. First, it is possible that the Sternberg and Stroop task might not be affected by cerebellar tDCS, though there is literature suggesting an effect of cerebellar tDCS over the cerebellum on working memory in particular [44,45]. But, to the best of our knowledge, there is no evidence of cerebellar tDCS modulating performance for executive function tasks, such as the Stroop task. Furthermore, it is important to note a recent meta-analysis that suggests the effects of cerebellar tDCS might be task specific. That is, the current body of work seems to suggest that cerebellar tDCS is more effective on motor tasks than on cognitive tasks [2]. It should also be noted that the number of studies using tDCS to modulate cognitive task performance is limited, perhaps making it difficult to see the true effect of stimulation in individual cognitive domains. Thus, it may be the case that the possibility of task specific effects of cerebellar tDCS are related to the null findings from the Stroop task; however, this is likely not the case for the Sternberg task given past work to the contrary [43–46]. Additional future work investigating the task specificity of cerebellar tDCS is warranted to better understand the boundary conditions and limitations of this methodology.

Second, task difficulty is a key consideration. In general, for healthy young participants, as recruited here, these tasks are relatively easy. The average accuracy score across participants was over 90%, 75% of the time (see Table 2). It is possible that the tasks were so easy for these participants that even if tDCS disrupted cerebellar function, other cortical brain regions, like the prefrontal cortex, were able to handle the load and complete the task without the aid of cerebellar contributions. Thus, future work would benefit from understanding whether task difficulty influences the effectiveness of cerebellar tDCS. Notably, Pope & Miall (2012) found that cathodal stimulation over the cerebellum improved performance when participants were under high load. Additionally, the effects of stimulation were greater on tasks rated as difficult [43]. Although it might not be easily implemented for all tasks, task difficultly might be an important consideration when applying cerebellar tDCS to modulate performance.

Third, electrode size and placement might be a contributing factor to the current null findings. Traditionally, a two-electrode system [51] allows one large consistent stream of current to be applied to a brain region; however, this methodology, particularly as it is applied to the cerebellum, does not always produce consistent results [2], presumably due in part to its non-focal nature. To overcome this potential limitation, the HD-tDCS system was used here to improve targeting and increase the focality of stimulation, which in turn should increase the effect of stimulation. However, we did not see such an effect here. This might be due in part to the location of the cerebellum, tucked under the brain requiring the current to travel through more bone [83] and head fat [84] than for the cerebrum. Further, because the current is spread across nine electrodes, this reduces the strength of the current entering the cerebellum from each electrode. While the effective stimulation is equivalent to 2mA, there is less current coming from each source. Thus, individual differences in head characteristics and a reduction in current intensity may have caused the current to dissipate more quickly, reducing the effectiveness of the stimulation. Second, the cytoarchitecture of the cerebellum is substantially different from the cerebrum [27], thus current density and polarity patterns that might work in other brain regions might not be effective in the cerebellum, possibly resulting in the mixed findings we see in the cerebellar tDCS literature (c.f. [2]). For instance, because of the uniform nature and the consistent cell structure, it is possible that current levels and densities that excite Purkinje fibers in the cerebellum will be different from the levels that excite pyramidal neurons in the primary motor cortex [85]. Critically, with more focal stimulation, we still find null results. Therefore, future work might investigate how different levels of current impact different cells types and whether distance from the electrode and duration of stimulation have any moderating effects.

A fourth possible limitation, not mentioned by Steiner and colleagues [50], is that the current work used a single session of cathodal stimulation. Though past work using a single session of stimulation has found an effect of stimulation on task performance [43–46], other studies did not find such an effect [47–50]. This inconsistency might be because one tDCS session is not enough time for the effects of stimulation to take place. Multi-session tDCS studies have been successful in modulating task performance and might be a more effective way of facilitating changes in task performance as they are believed to induce long lasting effects, by modulating cortical plasticity [86]. Benussi et al. (2017) used this approach in an effort to reduce the performance deficits experienced by individuals with neurodegenerative ataxia [87]. The authors found that when 2 mA of anodal tDCS was applied for 20 minutes once a day for ten days within a two-week period, patients saw improved clinical scores and restored cerebellar brain inhibition pathways, compared to sham. Further, in healthy young adults, Meinzer and colleagues [88] applied 2 mA of anodal cerebellar stimulation for 20 minutes once a day for five days and improved language learning [88]. It is possible that with only one tDCS session, effects of stimulation are minimal, while multiple sessions allow for larger and more long-lasting effects of stimulation to manifest. Future work might investigate the benefit of multiple session of cerebellar tDCS on cognition.

While our current findings are not significant, they are important in furthering our understanding of a mixed, but growing cerebellar tDCS literature [2]. There is already evidence that not all attempts to use tDCS over the cerebellum are effective [47,48,50], and it is important to understand why. Notably, our extension of this literature through the use of more targeted HD-tDCS did not produce a significant effect of stimulation. Importantly, this indicates that more focal stimulation might not be an important factor in cerebellar tDCS. Future work might then focus how differences in intensity and polarity of stimulation affect the effect of stimulation in an effort to refine cerebellar tDCS procedures. However, more effort should also be made to understand whether HD-tDCS is a viable option for stimulation in both research and remediation, as the current work, to the best of our knowledge, is the first to use HD-tDCS to modulate cerebellar activity in the context of non-motor cognitive paradigms. Similarly, the file drawer effect [89] might hinder the broader understanding of the effect of tDCS on the cerebellum should null findings not be published. Because this technique holds promise for rehabilitation and treatment across a variety of diseases and infarcts [79,90,91] a clear understanding of the boundary conditions under which tDCS is effective (or not) in healthy adult populations is necessary so that stimulation parameters may be optimized in the future. There seems to be success using tDCS to treat many motor disorders such as stroke, Parkinson’s disease, multiple sclerosis and cerebral palsy (for a review see [92]). However, little work has looked to treat cognitive impairments, most likely because there is not enough understanding of how tDCS affects the cerebellum during cognitive tasks. The current study provides another data point in understanding how cerebellar tDCS should be applied to effectively modulate cognitive performance.

### Limitations

The current study did not have any sensorial surveys to judge whether participants knew what condition they completed. This was done for two related reasons. First, we did not want people relying on recollection when making a judgement about their first visit. Second, if we asked this question during the first visit, participants would potentially be unblinded to the stimulation condition and this might alter the way people complete the task. However, work by [93] suggest that participants are generally unaware of which stimulation condition they are in, even if they are aware a sham condition is possible. Thus, we believe this is the case in our current study.

### Conclusion

The current work aimed to better understand the role of the cerebellum in higher order cognition, through the use of HD-tDCS. However, we did not find an effect of stimulation. Importantly, this extends our understanding of the effects of tDCS on the cerebellum with respect to cognitive task performance. While we initially predicted that our HD montage would increase the effects of stimulation, this was not the case. Together, our findings provide additional evidence to suggest that the effects of tDCS on cerebellar contributions to cognition are mixed. Critically however, this does not discount the purported role of the cerebellum in cognition. We suggest that future work carefully consider task parameters such as task difficult in addition to stimulation parameters to that we may better understand the potential utility of this methodology for modulation of cerebellar function.

## Supporting information

S1 Table Sternberg data. (CSV)

S2 Table Stroop data. (CSV)
